# Characteristics of Ang-(1–7)/Mas-Mediated Amelioration of Joint Inflammation and Cardiac Complications in Mice With Collagen-Induced Arthritis

**DOI:** 10.3389/fimmu.2021.655614

**Published:** 2021-05-17

**Authors:** Zhongjie Wang, Wenhan Huang, Feifeng Ren, Lei Luo, Jun Zhou, Dongmei Huang, Mei Jiang, Huaan Du, Jinqi Fan, Lin Tang

**Affiliations:** ^1^ Department of Rheumatology and Immunology, Second Affiliated Hospital of Chongqing Medical University, Chongqing, China; ^2^ Department of Cardiovascular, Second Affiliated Hospital of Chongqing Medical University, Chongqing, China

**Keywords:** angiotensin-(1–7), Mas receptor, arthritis, cardiac complications, mice

## Abstract

**Objectives:**

Rheumatoid arthritis (RA) is a disabling disease with a high incidence that is regularly accompanied by cardiovascular complications. Several studies have suggested that renin–angiotensin–aldosterone system (RAAS) is closely associated with RA. The aim of this study was to investigate the mechanisms underlying Angiotensin-(1–7) [Ang-(1–7)] and its Mas receptor agonist (AVE0991) on joint inflammation and cardiac complications in a collagen-induced arthritis (CIA) model.

**Methods:**

Collagen type II was injected into DBA/1 mice to construct an arthritis model. CIA mice were treated with Ang-(1–7) (2.0 mg/kg intraperitoneally) and AVE0991 (3.0 mg/kg intraperitoneally). The serum levels of inflammatory cytokines [tumor necrosis factor-alpha (TNF-α), interleukin (IL)-1 β, IL-6, and C-reactive protein (CRP)] were determined by ELISA. The mitogen-activated protein kinase (MAPK) and nuclear factor-kappaB (NF-*κ*B) signaling pathways in joint tissues and the transforming growth factor (TGF)-*β*/Smad pathway and levels of *α*-Smooth muscle action (SMA) and *β*-myosin heavy chain (MHC) protein expression in cardiac tissues were assessed by western blots. The levels of TGF-*β*/Smad pathway, *α*-SMA, and *β*-MHC RNA in cardiac tissues were analyzed by real time-PCR. The levels of receptor activator of nuclear factor kappa ligand (RANKL) and promoting matrix metalloproteinase (MMP)-3 expression in the ankle joints were detected by immunohistochemistry and real time-PCR.

**Results:**

Ang-(1–7) and AVE0991 reduced the levels of inflammatory cytokines and inhibited the MAPKs and NF-*κ*B signaling pathways in ankle joint tissues, reduced RANKL and MMP3 expression, and ameliorated local joint inflammation and bone destruction compared with the control group. In addition, Ang-(1–7) and AVE0991 attenuated the TGF-*β*/Smad signaling pathway, reduced the levels of *α*-SMA and *β*-MHC expression, and diminished inflammatory cell infiltration into the myocardial interstitium and myocardial interstitial fibrosis in the hearts of CIA mice.

**Conclusions:**

Ang-(1–7) alleviated joint damage caused by inflammation likely through the attenuation of NF-*κ*B and MAPK pathways and ameliorated inflammation-induced cardiac fibrosis and activation of the TGF-*β*/Smad pathway. Moreover, Ang-(1–7) was likely mediated through the Mas receptor. This study provides theoretical evidence for exploring novel clinical therapeutic approaches for RA and its cardiac complications.

## Introduction

Rheumatoid arthritis (RA) is a chronic, systemic, autoimmune disease that is mainly characterized by proliferation of synovial cells and erosion of articular cartilage and bone (1). In recent years, it was found that the incidence of cardiovascular complications is significantly higher in RA patients than their peers (2). During the initiation and progression of RA, the articular synovial tissues are infiltrated with inflammatory cells, and subsequently secrete inflammatory cytokines, such as TNF-α, IL-1, IL-6 (3). A previous study has shown that the anti-arthritic effect is produced *via* inhibiting TNF-α and IL-1 and up-regulating anti-inflammatory mediators in the knee tissue of CIA mice (4). IL-6 activates inflammatory cells to enter the synovial membrane and propagate the inflammatory response (5), so inhibition of IL-6/IL-17A can potentially reduce joint inflammation and cartilage destruction of RA onset (6). Inflammatory cytokines induce the expression of RANKL *via* the NF-*κ*B (7) and MAPKs (8) pathways. Increased NF-*κ*B expression is also involved in several immune mediated inflammation (9). Notably, the inhibition of NF-*κ*B activity suppresses joint inflammation in mouse model of CIA (6). Therefore, MMP production by synovial fibroblasts leads to articular cartilage destruction and joint deformity (10). In contrast, inflammatory factors activate the TGF-*β*/Smad signaling pathway, which enhance the production of collagen and extracellular matrix by myocardial fibroblasts thus, leading to myocardial fibrosis (11).

The progression of RA and cardiac complications are thought to be closely associated with the renin–angiotensin–aldosterone system (RAAS) (12). Existing research has revealed that persistent activation of the RAAS can enhance proliferation and differentiation through the classic ACE/Ang–II/AT1R signaling pathway, resulting in joint damage in RA patients (13). Furthermore, persistent activation of the RAAS can also lead to myocardial hypertrophy, extracellular matrix accumulation, myocardial interstitial fibrosis, and other manifestations of ventricular remodeling (14).

In addition to the ACE/Ang–II/AT1R axis in the RAAS, another axis displays negative regulation effects (the ACE2/Ang-(1–7)/Mas axis). The binding of Ang-(1–7) in the axis and the Mas receptor distributed in joints, hearts, and blood vessels inhibits leukocyte aggregation, the expression and secretion of inflammatory cytokines, and the occurrence of organ fibrosis in inflammatory states (15). Ang-(1–7) and AVE0991 have been shown in an antigen-induced arthritis rat model to reduce neutrophil aggregation, and TNF-α and IL-1β expression in joint tissues (16). Ang-(1–7) inhibits inflammation and protects endothelial cells in a murine model of atherosclerosis, thereby impeding atherosclerosis (17). Thus, the ACE2/Ang-(1–7)/Mas axis has important effects on attenuating the pro-inflammatory and fibrotic processes in the RAAS, but the specific signaling pathway has not been elucidated. In the current study, CIA mice were used as a research model to investigate the elusive characteristics of disease inhibition of Ang-(1–7) and AVE0991 on joint inflammation and cardiac complications.

## Materials and Methods

### Animal Model and Group Division

The CIA model was established as follows. Avian type II collagen (Sigma, St. Louis, MO, USA) was mixed with an equal volume of complete Freund’s adjuvant (Sigma) to generate an emulsion mixture (1 mg of avian type II collagen and 2 mg of inactivated *Mycobacterium tuberculosis*/ml), 0.1 ml of which was injected into the tail base. Twenty-one days after the initial injection, the same volume of avian type II collagen was mixed with an equal volume of incomplete Freund’s adjuvant (Sigma) to generate an emulsion mixture, 0.1 ml of which was injected into the tail base subcutaneously to boost the immune response.

Sixty male DBA/1 mice, 7 weeks old, were purchased from the Laboratory Animal Center of Chongqing Medical University. The mice were housed in the animal center under SPF standards (temperature, 22–24°C; light, 6:00–18:00; humidity, 50–70%) with free access to food. The 60 mice were randomly divided into six groups with 10 mice in each group, as follows: 1) control, intraperitoneal injection of normal saline once daily for 1 week from day 28; 2) CIA, the CIA model was established based on the above described methods, with normal saline injected intraperitoneally once daily for 1 week from day 28; 3) CIA + Ang-(1–7), the CIA model was established using the previously described methods, with Ang-(1–7) (2.0 mg/kg) injected intraperitoneally once daily for 1 week from day 28; 4) CIA + AVE0991, the CIA model was established according to the above-described methods, with AVE0991 (3.0 mg/kg) injected intraperitoneally once daily for 1 week from day 28; 5) Ang-(1–7), Ang-(1–7) (2.0 mg/kg) was injected intraperitoneally once daily for 1 week from day 28; and 6) AVE0991, AVE0991 (3.0 mg/kg) was injected intraperitoneally once daily for 1 week from day 28. On day 42, all mice were sacrificed under anesthesia. This animal study was approved by the Animal Ethics Committee of the Second Affiliated Hospital of Chongqing Medical University, and the experimental procedures complied with the guidelines for the use of laboratory animals.

### Paw Thickness Measurement and Arthritic Index Scoring

From day 24, the thickness of the hind paws of mice was measured with Vernier calipers every 4 days. The inflammatory scores of all four paws were assessed using the following methods: 0 = normal; 1 = erythema or slight swelling in the ankle joints; 2 = erythema and slight swelling from the ankle joints-to-the toe or metacarpal joints; 3 = erythema and moderate swelling from the ankle joints-to-the toe or metacarpal joints; and 4 = erythema and severe swelling from the ankle joints-to-the toe or metacarpal joints. The score for each mouse was the sum of four paws; the minimum score was 0, and the maximum score was 16 (18). The arthritic index was assessed by two independent researchers.

### Specimen Collection

On day 42, retro-orbital blood was collected from mice under anesthesia. Serum was collected after centrifugation and stored at -80°C. The ankle joints and hearts were collected and stored in liquid nitrogen or fixed in paraformaldehyde.

### Serum Cytokine Levels by ELISA

Wells were designated for blank controls, standards, and samples for testing; and each sample was tested in duplicate. One hundred microliters of each sample was added to the designated wells. After incubation at room temperature, 100 μl of enzyme-labeled primary antibody solution was added to each well (except the blank control wells). Substrate for color development was added to each well and allowed to react in the dark for 15 min; the reaction was terminated by adding stop solution. The desired cytokine concentrations were determined using a standard curve, and the absorbance of standards, CRP, TNF-α, IL-1β, and IL-6 were measured at 450 nm (ELISA kit; Beijing Sizhengbai Technology Co., Ltd., Beijing, China).

### Protein Expression in Ankle Joints and Hearts Detected by Western Blots

The expression of p-p38, p38, p-ERK, ERK, p-JNK, JNK, NF-*κ*B, and I*κ*B-*α* protein in the ankle joint tissues and the expression of TGF-*β*1, Smad3, *α*-SMA, and *β*-MHC protein in cardiac tissues were detected *via* western blots. Phosphorylated proteins in tissues were extracted using a phosphoprotein extraction kit, and an equal volume of protein buffer was added to the extracted sample. The extracted protein sample from the tissues (20 µl) and the protein standard marker (5 µl) were loaded into the wells of an SDS-PAGE gel. The proteins in the SDS-PAGE gel were separated, followed by transfer to a PVDF membrane using an electrophoresis apparatus. The PVDF membrane was placed in blocking solution (5% BSA + TBST) and incubated for 1 h. The PVDF membrane was then incubated overnight with rabbit anti-mouse polyclonal antibody against the corresponding protein at 4°C. The PVDF membrane was washed with TBST and incubated with shaking in horseradish peroxidase-labeled IgG for 1 h. The protein bands on the PVDF membrane were exposed using an enhanced chemiluminescence (ECL) kit (KeyGEN Bio, China). The p-p38, p38, p-ERK, ERK, p-JNK, JNK, NF-*κ*B, and I*κ*B-*α* levels in the ankle joint tissues of mice and the TGF-*β*1, Smad3, *α*-SMA, and *β*-MHC levels in cardiac tissues were determined using a ChemiDoc imaging system (Bio-Rad Laboratories, Hercules, CA, USA).

The concentration configurations of relevant rabbit anti-mouse polyclonal antibodies were as follows: p-p38 (1:1,000; CST, USA); p38 (1:1,000; CST, USA); p-ERK (1:2,000; CST, USA); ERK (1:2,000; CST, USA); p-JNK (1:2,000; CST, USA); JNK (1:2,000; CST, USA); NF-*κ*B (1:2,000; CST, USA); I*κ*B-*α* (1:1,000; CST, USA); TGF-*β* (1:1,000; CST, USA); Smad3 (1:1,000; CST, USA); *α*-SMA (1:1,000; CST, USA); *β*-MHC (1:1,000; Abcam, USA); GAPDH (1:2,000; SAB, USA).

### RNA Expression in Ankle Joints and Hearts Based on Real-Time PCR

RNA was extracted from the ankle joints and heart tissues using an RNA extraction kit (Bioteke Corporation, China). RNA was reverse-transcribed into DNA using a RT reverse transcription kit (TaKaRa, Japan). Quantitative analysis was performed using GoTaq^®^ qPCR Master Mix (Promega Corporation, China) and a Bio-Rad CFX96 Real-time PCR Detection System. The RT-PCR conditions were as follows: a 10-min cycle at 95°C; 40 cycles for 15 s at 95°C; and a 60-s cycle at 60°C.The results were recorded according to the CT values displayed by the software, and the results were subsequently analyzed using the software. The 2^¯△△CT^ method was used for calculations. The primers used in the experiments are shown in **Table 1**.

**Table 1:** qRT-PCR primer sequences.

Gene	Primer sequences
GAPDH	F-AAATGGTGAAGGTCGGTGTGA R-GGCTTCCCGTTGATGACAAG
RANKL	F-TACTTTCGAGCGCAGATGGAT R-TGAGGTGTGCAAATGGCTGG
MMP-3	F-TTGTGTGCTCATCCTACCCA R-AAGCCACCAACATCAGGAACA
*α*-SMA	F-CCTTTCCACAGGGCTTTGTTTG R-CCAGTTCCTTCATTCTGCACTCG
*β*-MHC	F-CCAGTGTACAATGCGCAAGTG R-TGATAGGCGTTGTCAGAGATGG
TGF-*β*	F-TGTGGAGCAACATGTGGAACTCT R-TTGGTTCAGCCACTGCCGTA
Smad3	F-ATGTCATCTACTGCCGCTTGT R-GTCGCTAGTTTCTCCATCTTCAC

### Immunohistochemistry

The paraffin tissue sections of the ankle samples were placed in a 60°C constant temperature incubator and incubated for 120 min. The sections were dehydrated in an ethanol series with a gradient concentration, followed by deparaffinization in xylene. The sections were immersed in antigen retrieval solution and boiled for 20 min, followed by thrice-washing in PBS. After air-drying the section, goat blocking serum was added dropwise, and the primary antibodies against corresponding proteins were then added dropwise. The sections were incubated overnight in the dark at 4°C. On the next day, after washing the sections with PBS, secondary antibodies were added dropwise, and the sections were incubated at room temperature for 20 min. DAB chromogen solution (ZSGB-bio, China) was added, and the tissue sections were re-stained with hematoxylin for 3 min. The sections were then rinsed thoroughly with tap water before mounting with coverslips. Immunohistochemical staining sections were observed and photographed using an image analysis system (NIS Elements). ImageProPlus6.0 software was used for semi-quantitative analysis of positively stained areas in ankle joint tissues and the IOD/area values were calculated.

The concentration configurations of antibodies against corresponding proteins were as follows: anti-MMP-3 (1:50; Arigo, Taiwan); and anti-RANKL (1:100; Arigo, Taiwan).

### H&E Staining

The ankle joints and hearts were fixed, then the joint tissues were decalcified and embedded in paraffin to produce tissue sections for hematoxylin and eosin (H&E) staining. The H&E-stained sections were inspected and photographed using an image analysis system (NIS Elements; Nikon, Sendai, Japan). Ankles were evaluated as follows: synovial proliferation; inflammatory cell infiltration; pannus formation; and bone destruction (19). The score for each parameter ranged from 0 to 3 points. The score was independently assessed by two pathologists.

### Masson Staining of the Heart

The cardiac tissues were fixed and paraffin-embedded to produce tissue sections for Masson staining. Masson-stained sections were observed and photographed using an image analysis system (NIS Elements). Blue staining was positive. ImageProPlus6.0 software was used for semi-quantitative analysis of the positive staining signals in cardiac tissues, and the IOD/area values were calculated.

### Statistical Analysis

Statistical analysis was performed on all data using SPSS Statistics 20.0 software. One-way ANOVA test was used to analyze data that was normally distributed and the Kruskal–Wallis test was used to analyze data that were not normally distributed (arthritic and joint histopathology scores). A p <0.05 was considered statistically significant.

## Results

### Ang-(1–7) and AVE0991 Reduced the Inflammatory Cytokine Expression in the Serum of Mice With CIA

**Figure 1:**
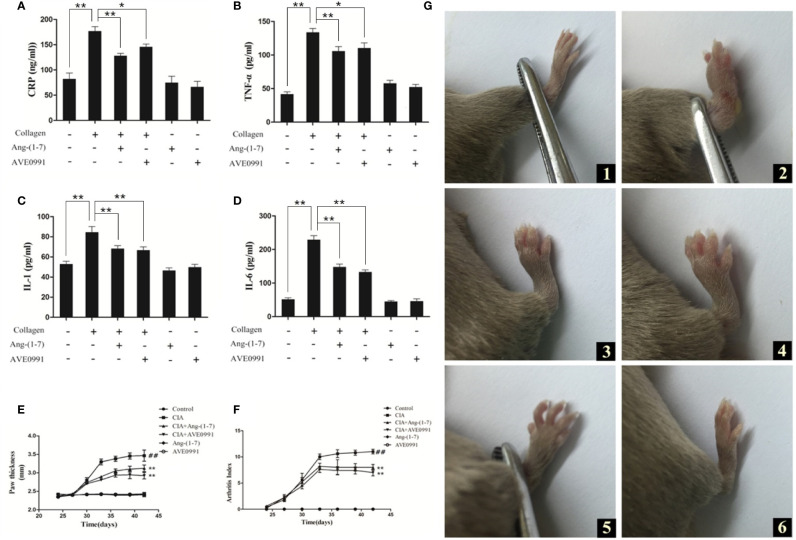
Effect of Ang-(1–7) and AVE0991 on development of inflammatory factor in mice. The serum cytokine levels of **(A)** CRP, **(B)** TNF-α, **(C)** IL-1β, and **(D)** IL-6 were determined using ELISA kits. Values are expressed as means ± SD (n = 6). *P < 0.05, **P<0.01. Effect of Ang-(1–7) and AVE0991 on development of hind paw thickness and Arthritis Index in CIA mice.**(E)** The hind paw thickness of DBA/1 mice was measured every three days in all groups from day 24. Values are expressed as means ± SD (n = 10). **(F)** The Arthritis Index of DBA/1 mice were measured every three days in all groups from day 24. Values are expressed as means ± SD (n = 10). **: Compared with CIA group, P<0.01; ##: Compared with Control group, P<0.01. Paw swelling in mice. **(G)** (1) Control group, (2) CIA group, (3) CIA + Ang-(1**–**7) group, (4) CIA + AVE0991 group, (5) Ang-(1**–**7) group, (6) AVE0991 group.

The CRP, TNF-α, IL-1β, and IL-6 levels in the serum of mice with CIA were significantly higher than those in the control group (**Figures 1A–D**). The CRP, TNF-α, IL-1β, and IL-6 levels in the serum of CIA+ Ang-(1–7) and CIA + AVE0991 mice were significantly reduced compared to the CIA group (**Figures 1A–D**).

### Ang-(1–7) and AVE0991 Relieved Swelling in the Paws of CIA Mice and Reduced the Arthritic Index

On day 42, the mouse paws exhibited notable swelling in the CIA group. The swelling in the CIA + Ang-(1**–**7) and CIA + AVE0991 groups was markedly less compared to the CIA group (**Figure 1G**). The paw thickness and arthritic index of mice in the CIA group were significantly greater than those in the control group, while the paw thickness and arthritic index of mice in the CIA + Ang-(1**–**7) and CIA + AVE0991 groups were significantly lower than those in the CIA group (**Figures 1E, F**).

### Ang-(1–7) and AVE0991 Reduced Histologic Joint Damage in CIA Mice

**Figure 2:**
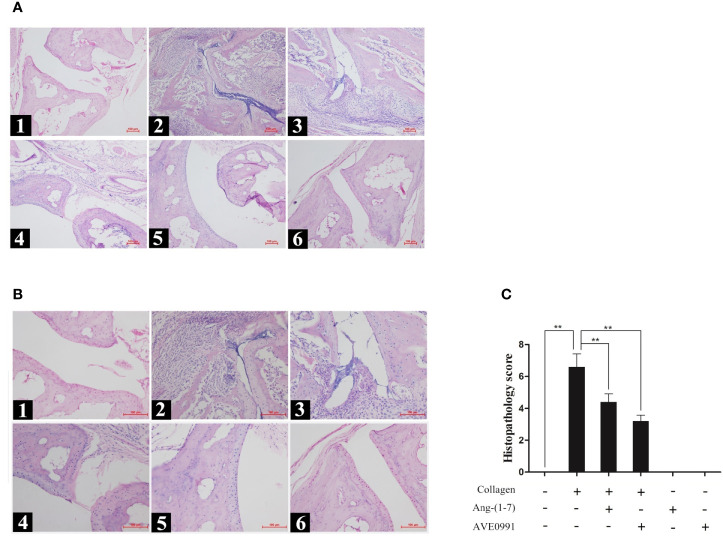
H&E staining of ankle in mice. (A1, B1) Control group, (A2, B2) CIA group, (A3, B3) CIA + Ang-(1–7) group, (A4, B4) CIA + AVE0991 group, (A5, B5) Ang-(1–7) group, (A6, B6) AVE0991 group. Magnification =100× **(A)** and 200× **(B)**. **(C)** The ankle pathological score was graded in a blinded manner. Values are expressed as means ± SD (n = 6), **P<0.01.

Mice in the CIA group exhibited synovial tissue hyperplasia and articular cavity invasion, notable joint space narrowing, extensive inflammatory cell infiltration, and remarkable bone destruction. The histopathologic score of the ankle joint in the CIA group was significantly higher than that in the control group. In contrast to the CIA group, mice in the CIA + Ang-(1**–**7) and CIA + AVE0991 groups showed markedly reduced synovial tissue hyperplasia, inflammatory cell infiltration, and bone destruction (**Figures 2A, B**). The scores in the CIA + Ang-(1**–**7) and CIA + AVE0991 groups were significantly lower than those in the CIA group (**Figure 2C**).

### Ang-(1–7) and AVE0991 Reduced the Expression of RANKL and MMP-3 in the Ankle Joints of Mice With CIA *via* the MAPKs and NF-κB Pathways

**Figure 3:**
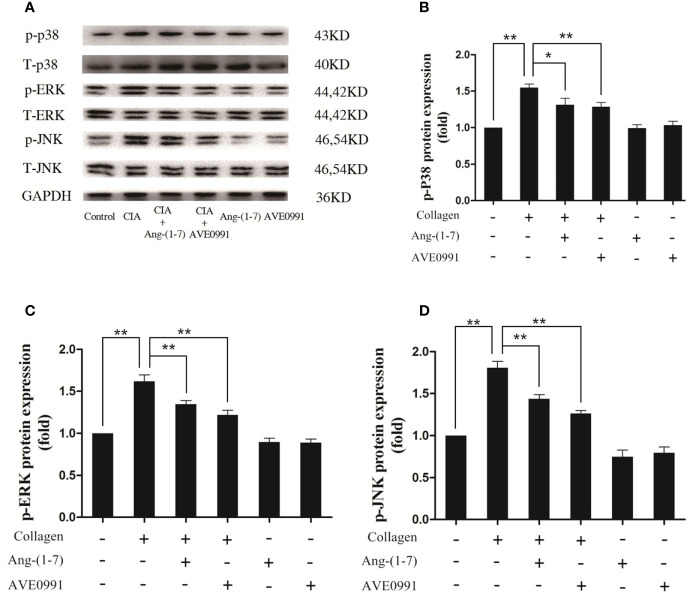
Ang-(1–7) and AVE0991 inhibit arthritis-induced p-p38, p-ERK, and p-JNK expression in ankle. **(A)** Representative immunoblots for p-p38、p38、p-ERK、ERK、p-JNK, and JNK. **(B)** Representative quantitative analysis of p-p38. **(C)** Representative quantitative analysis of p-ERK. **(D)** Representative quantitative analysis of p-JNK. Values are expressed as means ± SD (n = 6). *P < 0.05, **P<0.01.

**Figure 4:**
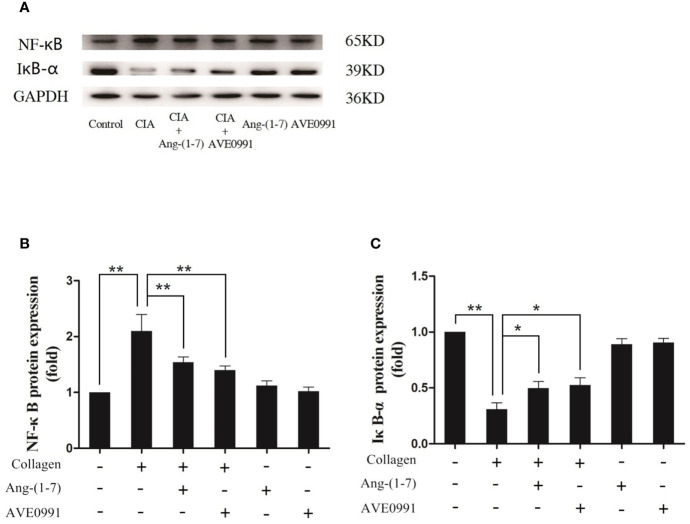
Ang-(1–7) and AVE0991 inhibit arthritis-induced NF-*κ*B and increase arthritis-induced I*κ*B-*α* expression in the ankle. **(A)** Representative immunoblots for NF-*κ*B and I*κ*B-*α*. **(B)** Representative quantitative analysis of NF-*κ*B. **(C)** Representative quantitative analysis of I*κ*B-*α*. Values are expressed as means ± SD (n = 6). *P < 0.05, **P < 0.01.

Joint destruction was clearly demonstrated in mice with CIA. Ang-(1**–**7) and AVE0991 had protective effects on joints. To further explore the mechanism of action underlying Ang-(1**–**7) and AVE0991, we used western blots to evaluate the expression of p-p38, p-ERK, and p-JNK proteins in the ankle joint MAPK pathway, and the expression of NF-*κ*B and I*κ*B-*α* proteins in the ankle joint NF-*κ*B pathway. The expression of p-p38, p-ERK, and p-JNK protein in the ankle tissues of the CIA group significantly increased (**Figures 3B–D**), and the expression of I*κ*B-*α* in the ankle tissues of the CIA group significantly decreased compared to that of the control group (**Figure 4C**). The expression of NF-*κ*B in the ankle tissues of CIA + Ang-(1**–**7) and CIA + AVE0991 mice was significantly decreased (**Figure 4B**), and the expression of I*κ*B-*α* in the ankle tissues of CIA + Ang-(1**–**7) and CIA + AVE0991 mice was significantly increased compared to that of mice with CIA (**Figure 4C**).

**Figure 5:**
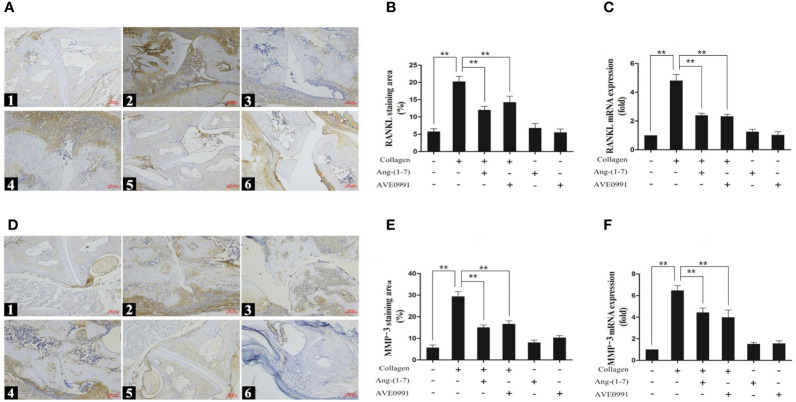
Immunohistochemical staining and nucleic acid expression of RANKL and MMP-3 in ankle. (A1, D1) Control group, (A2, D2) CIA group, (A3, D3) CIA + Ang-(1–7) group, (A4, D4) CIA + AVE0991 group, (A5, D5) Ang-(1–7) group, (A6, D6) AVE0991 group. Magnification = 100× **(A, D)**. **(B)** Quantification of RANKL staining evaluated with Image-Pro Plus v 6.0. **(E)** Quantification of MMP-3 staining evaluated with Image-Pro Plus v 6.0. The mRNA expression of **(C)** RANKL and **(F)** MMP-3 was detected by real-time PCR. Relative gene expression was calculated using the 2^¯△△CT^ method; the GAPDH gene served as the internal control. Values are expressed as means ± SD (n = 6), **P < 0.01.

Immunohistochemistry and real-time PCR were used to further determine the expression of skeletal damage markers (RANKL and MMP-3) downstream of the bone MAPK and NF-*κ*B pathways. Compared to the control group, the stained areas of RANKL and MMP-3 expression in the ankle joints of mice with CIA were significantly expanded, and there was a significant increase in nucleic acid expression. Ang-(1**–**7) and AVE0991 significantly reduced the expression of RANKL and MMP-3 in the ankle joints of mice with CIA (**Figure 5**).

### Ang-(1–7) and AVE0991 Ameliorated the Histopathologic Changes of Cardiac Tissues in Mice With CIA

**Figure 6:**
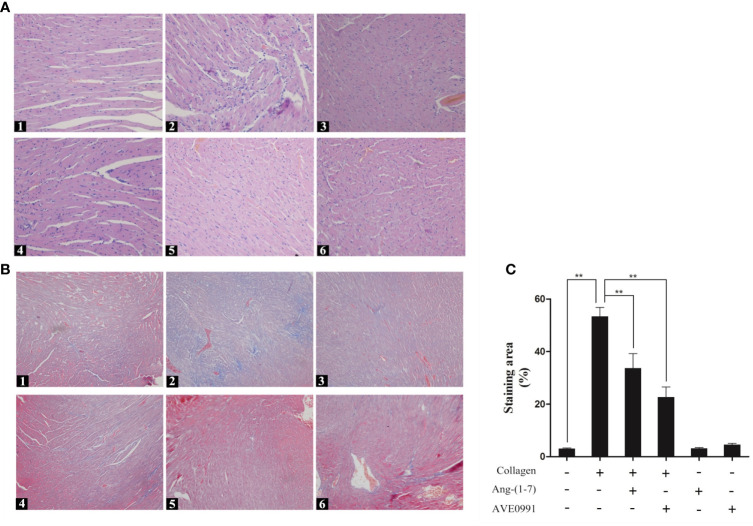
H&E staining of heart in mice. **(A1)** Control group, **(A2)** CIA group, **(A3)** CIA + Ang-(1**–**7) group, **(A4)** CIA + AVE0991 group, **(A5)** Ang-(1**–**7) group, **(A6)** AVE0991 group. Magnification = 200×. Masson staining of heart in mice. Representative Masson staining of heart in DBA/1 mice in all groups. **(B1)** Control group, **(B2)** CIA group, **(B3)** CIA + Ang-(1**–**7) group, **(B4)** CIA + AVE0991 group, **(B5)** Ang-(1**–**7) group, **(B6)** AVE0991 group. **(C)** Quantification fibrous staining region evaluated with Image-Pro Plus v 6.0. Values are expressed as means ± SD (n = 6). **P < 0.01.

Because the cardiac complications of RA were significantly increased, we needed to further explore the heart changes in CIA mice. H&E staining of the heart showed that the myocardial cells of mice in the control group were arranged in an orderly and compact manner, and the myocardial bundle morphology was intact. The myocardial cells of mice in the CIA group exhibited a disordered arrangement, and extensive inflammatory cells had infiltrated into the myocardial stroma. The inflammatory cell infiltration in the CIA + Ang-(1**–**7) and CIA + AVE0991 groups was significantly inhibited compared to that in the CIA group (**Figure 6A**). Masson staining of the heart showed that the myocardial tissue was red in the control group, and only a small amount of blue-stained fibrous connective tissue was present in the myocardial stroma. In the CIA group, normal myocardial tissue was replaced by a large area of fibrotic tissue. The blue-stained fibrous connective tissue area of the CIA + Ang-(1**–**7) and CIA + AVE0991 mice was significantly reduced compared to that of the CIA group (**Figures 6B, C**).

### Effects of Ang-(1–7) and AVE0991 Inhibited the Expression of TGF-β1, Smad3, α-SMA, and β-MHC in the Cardiac Tissues of Mice With CIA

**Figure 7:**
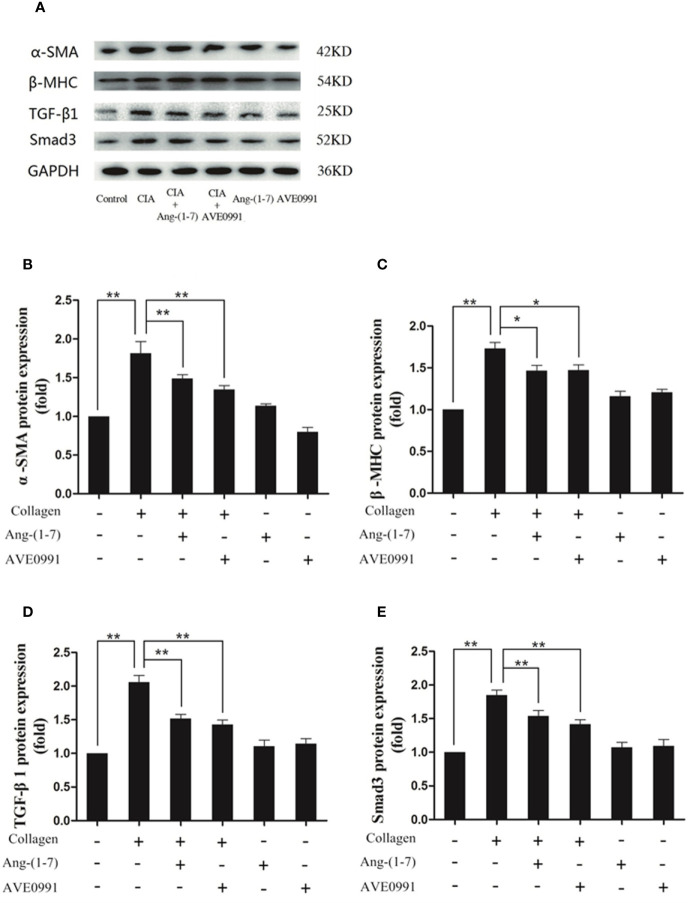
Ang-(1–7) and AVE0991 inhibit *α*-SMA、*β*-MHC、TGF-*β*1 and Smad3 protein expression in heart of CIA model. **(A)** Representative immunoblots for *α*-SMA、*β*-MHC、TGF-*β*1, and Smad3. **(B)** Representative quantitative analysis of *α*-SMA. **(C)** Representative quantitative analysis of *β*-MHC. **(D)** Representative quantitative analysis of TGF-*β*1. **(E)** Representative quantitative analysis of Smad3. Values are expressed as means ± SD (n = 6). *P < 0.05, **P < 0.01.

**Figure 8:**
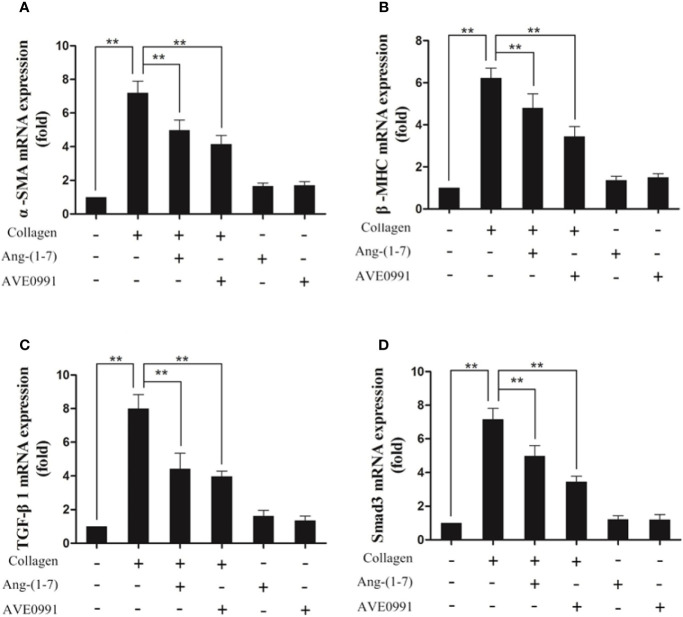
Ang-(1–7) and AVE0991 inhibit arthritis-induced *α*-SMA、*β*-MHC、TGF-*β*1, and Smad3 mRNA expression in the heart. The mRNA expression of **(A)** *α*-SMA, **(B)** *β*-MHC, **(C)** TGF-*β*1 and **(D)** Smad3 was detected by real-time PCR. Relative gene expression was calculated using the 2^¯△△CT^ method; the GAPDH gene served as the internal control. Values are expressed as means ± SD (n = 6), **P<0.01.

Because Ang-(1**–**7) and AVE0991 have protective effects on the hearts of CIA mice, we needed to further explore the underlying mechanism. Western blotting and RT-PCR results demonstrated that the expression of TGF-*β*1, Smad3, *α*-SMA, and *β*-MHC proteins, and RNA was significantly elevated in the cardiac tissues in the CIA group compared to that in the control group, and the expression of TGF-*β*1, Smad3, *α*-SMA, and *β*-MHC proteins and RNA was significantly decreased in the CIA + Ang-(1–7) and CIA + AVE0991 groups compared to that in the CIA group (**Figure 7**). Those data indicated that Ang-(1–7) and AVE0991 regulate the TGF-*β*/Smad pathway in cardiac tissues of CIA mice at the protein and mRNA levels, thus inhibiting myocardial fibrosis (**Figure 8**).

## Discussion

RA is a chronic, systemic, autoimmune disease that is primarily characterized by an inflammatory cell infiltrate in the joints, synovial cell hyperplasia, and bone erosion (1). In the early stage of the disease, inflammatory cells infiltrate the articular synovial tissues and subsequently secrete inflammatory cytokines, such as TNF-α, IL-1, and IL-6, after local activation to promote the initiation and progression of the disease (3). Our previous study showed that the ACE2/Ang-(1–7)/MAS axis is involved in the regulation of inflammation. Ang-(1–7) impedes the activation of TGF-*β*1 induced by LDL and subsequently protecting the human mesangial cells from injury by regulating the LDLr-SREBP2-SCAP pathway (20). Ang-(1–7) reduces pro-inflammatory cytokine secretion in the serum of mice in a state of inflammation under high-fat diet conditions, modulating lipid absorption in renal tissues by regulating the LDLr-SREBP2-SCAP pathway and as a result, alleviating pathologic injury and renal function (21). Ang-(1–7) regulates LPS-induced inflammatory responses *via* the TLR4/JNK/FoxO1 signaling pathway in macrophage inflammatory models (22). It was found that decreased mRNA levels of TNF-α in knee tissues could produce the anti-inflammatory effects in CIA mice (23). Based on the previous work, our study found that Ang-(1–7) significantly inhibited the levels of TNF-α, IL-1, IL-6, and serum CRP in mice, reduced the swelling of mouse joints, and reduced the arthritis index in mice treated with Ang-(1–7). Consequently, our experiments revealed that Ang-(1–7) is a powerful anti-inflammatory factor.

TNF-α, IL-1, and IL-6 are significant inflammatory cytokines in the pathogenesis of RA (3). These overexpressed inflammatory cytokines induce the expression of RANKL *via* the NF-*κ*B (7) and MAPK (8) pathways, thus promoting the activation of synovial fibroblasts and osteoclasts. The inhibition of NF-*κ*B activity reduces the production of pro-inflammatory cytokines and further modulates related inflammatory reactions (24, 25). The MMP produced by activating synovial fibroblasts and osteoclasts cleave proteoglycans and collagen fibers in articular cartilage, resulting in joint destruction (10). In the present study, the expression of p-p38, p-ERK, and p-JNK in MAPK pathway proteins in the ankle joints of mice with CIA was significantly elevated, the expression of I*κ*B-*α* in NF-*κ*B pathway protein was decreased, and the expression of NF-*κ*B in the NF-*κ*B pathway protein was increased. Hence, the MAPK and NF-*κ*B pathways were shown to participate in the initiation and progression of arthritis in mice with CIA. Furthermore, we observed that the MAPK and NF-*κ*B activation can induce the massive expression of downstream RANKL and MMP-3 in mouse joints and eventually lead to a large number of inflammatory cells infiltrating synovium and surrounding tissues, joint space narrowing, bone destruction, and joint swelling. Our study demonstrated for the first time that Ang-(1–7) reduced the expression of p-p38, p-ERK, p-JNK, and NF-*κ*B in the ankle tissues of CIA mice and promoted the expression of I*κ*B-*α*, which confirmed that Ang-(1–7) can inhibit the activation of MAPK and NF-*κ*B signaling pathway. At the same time, Ang-(1–7) decreased the production of RANKL and MMP-3, which mediated bone destruction and alleviated the infiltration of inflammatory cells, bone destruction, and joint swelling in CIA mice, thus reducing joint destruction.

Studies have revealed that the incidence of cardiovascular complications (ventricular hypertrophy, myocardial fibrosis, and coronary atherosclerosis) in RA patients are approximately 1.5-fold higher than peers without RA (26). Our study showed that H&E and Masson staining in the hearts of CIA mice showed the infiltration of inflammatory cells and the formation of a large amount of fibrous connective tissue, indicating the presence of heart involvement in CIA mice. Previous studies have shown that activation of the TGF-*β*/Smad pathway by inflammatory cytokines, such as TNF-α, IL-1β, and IL-6, can enhance the expression of the myocardial fibrosis biomarker, *α*-SMA, and myocardial hypertrophy biomarker, *β*-MHC, induce the production of collagen and extracellular matrix, and subsequently lead to conditions, such as myocardial hypertrophy, myocardial interstitial fibrosis, and ventricular remodeling (11, 27, 28). Our previous study demonstrated that in primary rat cardiac fibroblasts, the binding of Ang-(1–7) to the Mas receptor attenuated Ang-II-induced ERK activation and exerted an anti-fibrotic effect (29). Therefore, by treating CIA mice with Ang-(1–7), we found that the expression of TGF-*β*1, Smad3, *α*-SMA and *β*-MHC was downregulated, which significantly reduced the infiltration and fibrosis of myocardial interstitial inflammatory cells. Hence, we first confirmed that Ang-(1–7) improved the cardiac injury of CIA mice by regulating TGF-*β*/Smad pathway.

The Mas receptor is a G protein-coupled receptor. A number of *in vivo* and *in vitro* experiments indicated that Ang-(1–7) exerted biological effects by interacting with the Mas receptor (15–17). In the current study, a Mas receptor agonist was injected into mice with CIA intraperitoneally. The results showed that AVE0991 exhibited almost the same effect as Ang-(1–7). Therefore, it was verified that the protective function of Ang-(1–7) on joints and hearts of mice with CIA was likely *via* the Mas receptor.

In conclusion, we demonstrated that the attenuation of the MAPK and NF-*κ*B signaling pathways by Ang-(1–7) likely *via* the Mas receptor reduced inflammatory cytokine secretion, inhibited RANKL and MMP3 expression, and subsequently alleviated joint damage in mice with CIA. It was verified that Ang-(1–7) impeded the TGF-*β*/Smad pathway likely *via* the Mas receptor, hence reducing *α*-SMA and *β*-MHC expression, diminishing infiltration of cardiac stromal inflammatory cells and fibrosis, and alleviating cardiac injury in CIA mice. The current study demonstrated, for the first time, that Ang-(1–7) ameliorated joint inflammation and cardiac complications in mice with CIA, providing a theoretical evidence for exploring novel therapeutic approaches for RA and the associated cardiovascular complications.

## Data Availability Statement

The original contributions presented in the study are included in the article/supplementary material. Further inquiries can be directed to the corresponding author.

## Author Contributions

Experimental design and experimental guidance were contributed by LT. Experimental operation and thesis writing was contributed by ZW and WH. Image acquisition was contributed by FR and LL. Document arrangement was contributed by JZ. Data statistics was contributed by DH and MJ. Experimental design of cardiac complications was contributed by HD and JF. All authors contributed to the article and approved the submitted version.

## Funding

This study was supported by a grant 81771738 from the National Natural Science Foundation of China and a grant (2020) 7 from the Kuanren Talents Program of the second affiliated hospital of Chongqing Medical University.

## Conflict of Interest

The authors declare that the research was conducted in the absence of any commercial or financial relationships that could be construed as a potential conflict of interest.
